# HPV Infection in Cervical and Other Cancers in Saudi Arabia: Implication for Prevention and Vaccination

**DOI:** 10.3389/fonc.2014.00065

**Published:** 2014-03-31

**Authors:** Ghazi Alsbeih

**Affiliations:** ^1^Research Centre, King Faisal Specialist Hospital and Research Centre, Riyadh, Saudi Arabia

**Keywords:** human papillomavirus, HPV genotype, HPV-16, cervical cancer, Saudi Arabia

## Abstract

Human papillomavirus (HPV) is closely associated with cervical cancer that the incidence of this tumor is regarded as a surrogate marker for HPV infection in countries lacking epidemiological studies. HPV is also implicated in subsets of anogenital and oropharyngeal cancers. Although cervical cancer is the third most common cancer in women worldwide, its reported incidence is low in Saudi Arabia, ranking number 12 between all cancers in females and accounts only for 2.4% of all new cases, despite the lack of national screening programs. However, the limited available studies from Saudi Arabia indicate that HPV prevalence and genotypes’ distribution in invasive cervical cancer show similar pattern as in the world. Cytology screening (Pap smear) and HPV vaccinations are the two preventive measures against cervical cancer. The two available vaccines are effective against the two most common HPV genotypes (HPV-16 and -18). Since 92% of cervical tumors in the Kingdom are infected with HPV of which 78% are HPV-16 and -18 genotypes, vaccination is expected to protect against more than two-third of cervical cancers in Saudi Arabia. Nevertheless, due to its low incidence (2.1/100,000 women), a proper cost-effectiveness analysis is required to justify the implementation of a costly vaccine bearing in mind that HPV could potentially be associated with about 3% of all cancers. However, further studies are needed to ascertain the real prevalence of HPV at the population level at large, its association with various types of cancers, and also the impact of local tradition and emerging behavioral trends that could affect HPV transmission and consequently the effectiveness of applying national vaccination program.

## Introduction

Human papillomavirus (HPV) has been overwhelmingly associated with cervical cancer that the incidence of this malignancy is deemed as surrogate indicator of HPV infection in countries lacking epidemiological studies. This can easily be justified because the worldwide HPV prevalence in cervical cancer has been estimated between 85 and 99% (1, 2). According to the International Agency for Research on Cancer, report GLOBOCAN 2008, cervical cancer is the third most common cancer in women worldwide, and the seventh overall, with an estimated 530,000 new cases in 2008 (3). More than 85% of the global burden occurs in developing countries, where it accounts for 13% of all female cancers. Age standardized rates show that cervical cancer is the second most common cancer in women in less developed countries while it ranks number 7 in developed countries. This is due to the lack of proper screening program (4). Pap smear screening, which identifies cytological abnormalities of the cervical transformation zone, has helped reducing cervical cancer incidence and mortality rates by 70% in developed countries (5).

## HPV Infection and Related Cancers

Human papillomavirus infection is common particularly in sexually active period of age. Reported estimates of incident HPV infection among initially negative women have reached as high as 60% over a 5-year follow-up period (6). The population-wide prevalence of HPV in women varies among studies and countries from 1.5 to 39% and closely reflects age and sexual activity (7–9). In addition, HPV infection has also been implicated in other cancers such as 90% of anal cancers and a smaller subset (<50%) of oropharyngeal, penile, vaginal, and vulvar cancers. In total, HPV may account for 5.2% of the worldwide cancer burden (10).

In contrast to the global view, the incidence of cervical cancer is very low in Saudi Arabia, ranking number 12 between all cancers in females and accounts only for 2.4% of all new cases (11), despite the lack of national screening programs. The incidence rate is extracted from the Saudi Cancer Registry (SCR), which is a population-based registry developed in 1992. It was established under the jurisdiction of the Ministry of Health and commenced reporting cancer cases from 01 January 1994. Although it relies on the collaboration of 500 governmental and private hospitals, physician’s offices, cancer treatment centers, and pathology laboratories located throughout the country, full coverage of all cancer cases cannot be ascertained. Nevertheless, in view of the lack of national screening programs, the actual reason for this low incidence is unknown. The closed society and standards of mores could reduce women’s exposure to HPV infection (12–15). Although cervical cancer is both preventable and curable, due to the lack of accessible screening in Saudi Arabia, most cases are presented at advanced stages (16, 17), that require extensive chemo-radiation therapy. This is due to the lack of proper screening program (4).

Data concerning the prevalence of HPV infection, HPV genotypes, and its relationship with cervical cancer are globally scarce in Saudi Arabia. Studies profiling Pap smears examined at University Hospitals have revealed precursor lesions of cervical cancer (14, 18–20). A study combining HPV detection with Pap test in 100 women undergoing voluntarily cervical cytological screening have found 6% HPV-positive cases that consisted of 5% high-risk and 1% low risk HPV (21). This percentage has further been confirmed in a larger, more recent study including 485 women seeking general gynecologic care at King Abdulaziz University Hospital in western region of Saudi Arabia (22). In contrast to these low risk settings, a study performed on 120 women attending routine gynecological examination in a hospital-based community, with dominant human components that has international acquaintances, has reported a prevalence of 31.6% infection with HPV-16/18 (23). In this study, none of the seven subjects with abnormal cytology had progressed to cervical intraepithelial neoplasia (CIN3) after 4 years of follow-up; suggesting non-persisting infection (24, 25). However, these studies were all confined to communities revolving around major hospitals and had included limited number of subjects. In addition, as local tradition limits sexual activities to marriage with no studies of the impact of travel on infection transmission considering the large portion of Saudi young generation traveling abroad for studies, business, and leisure, the real epidemiological prevalence of HPV infection in native Saudi population is still unknown.

The magnitude of the association between HPV infections and genotypes as causative agent of cervical cancer has been recently evaluated in limited number of patients in two independent studies from the same institute (26, 27). The first study had included 100 paraffin-embedded cervical biopsies with histopathologically proven cervical cancer. By histology, 82% were squamous cell carcinoma and 18% were adenocarcinoma of the cervix. Eleven patients had reported other unidentified past cervical infections and only six had prior screening. HPV detection and genotyping was carried out using the Linear Array kit (Roche Diagnostic) that enables the concomitant detection of 37 mucosal HPVs including 13 most common high-risk viruses. Results showed that 89% were positive for HPV infection. By histopathology, 93% of squamous cell carcinomas and 72% of adenocarcinomas are HPV positive. In total, 11 different HPV genotypes were detected, 8 of which (16, 18, 31, 39, 45, 51, 59, 73) are commonly classified as high-risk (87%) and 3 (6, 64, and 70) are classified as low risk (2%) HPVs. Thus, the prevalence of high-risk genotypes was 97.8% of HPV-positive tumors, which is comparable to the regional results obtained by Darnel et al. involving 44 Syrian women with invasive cervical cancer (28). The frequencies of the different HPV genotypes detected are summarized in (Table 1).

**Table 1:** **Prevalence of different HPV genotypes in two studies including 190 (92% HPV positive) cervical cancer patients in Saudi Arabia**.

HPV genotypes	Classification	Prevalence (%)	
		Alsbeih et al. (26)	Al-Badawi et al. (27)
**SINGLE INFECTION**			
HPV-6	LR	1.1	
HPV-16	HR	65.2	63.4
HPV-18	HR	3.4	11.1
HPV-31	HR	7.9	2.2
HPV-33			3.3
HPV-45	HR	6.7	4.5
HPV-52			2.2
HPV-53			2.2
HPV-58			2.2
HPV-59	HR	1.1	2.2
HPV-64	LR	1.1	
HPV-66			2.2
HPV-73	HR	2.3	
**CO-INFECTIONS**			
HPV-16/18	HR/HR	6.7	
HPV-16/39	HR/HR	1.1	
HPV-16/51	HR/HR	1.1	
HPV-16/70	HR/LR	1.1	
HPV-45/59	HR/HR	1.1	

*LR, low risk; HR, high-risk*.

In agreement with other studies, the most common HPV genotype was HPV-16 (29) with a prevalence of 65.2% compared to 54.4% in the world (30). The following most common genotypes by decreasing prevalence were: HPV-31 (7.9%), HPV-45 (6.7%), HPV-18 (3.4%), and HPV-73 (2.3%). The HPV genotypes (6, 39, 51, 59, 64, and 70) had an estimated prevalence of 1.1% each. Co-infections implicated HPV-16 with HPV-18 (6.7%), HPV-39, HPV-51, and HPV-70 (1.1% each), and HPV-45/59 (1.1%). With double infections, the two most common HPV genotypes were 16 and 18 with an estimated overall prevalence of 70% of all patients and 78.7% of HPV-positive tumors. This is comparable to the prevalence observed in Europe (74.5%), North America (76.5%), and in the whole-world (70.9%). However, these results seem to be different from those obtained in another Middle Eastern country where the most common HPV genotype was 33, which was detected at low prevalence in one of the two referenced study in Table 1, followed by 16 and 18 (28). Interestingly, as it had been described in the literature (25, 31), the two most common HPV genotypes (16 and 18) were more frequent in younger age group, and caused cervical cancer to occur 5 years earlier than other HPV infected patients. Furthermore, age-specific HPV distribution in Saudi cervical cancer patients showed a bimodal curve with a first peak at younger ages (41–45 years) and a relative rebound at older ages (56–60 years) as it has been described in other population (32).

**Figure 1:**
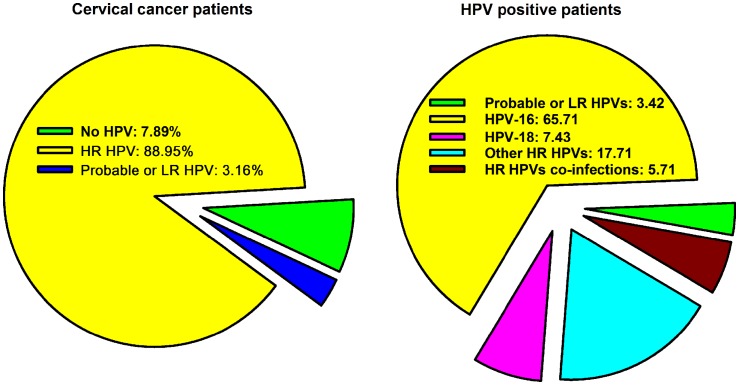
**The prevalence of HPV infection and genotypes distribution in cervical cancer in Saudi Arabia compiled from two published studies (26, 27)**.

The second study included 90 patients with cervical cancer and had essentially reached similar results using polymerase chain reaction amplification methods with two common primers, MY09, MY11 and GP5+, GP6+ that amplify a wide range of HPVs of which isolates were genotyped using DNA sequencing and reverse line blot hybridization assay to identify the high-risk HPV genotypes (27). Results showed that 95.5% were HPV positive. The most common HPV genotype detected was HPV-16 (63.4%), HPV-18 (11.1%), HPV-45 (4.5%), HPV-33 (3.3%), and HPV-31, HPV-52, HPV-53, HPV-58, HPV-59, and HPV-66 with 2.2% prevalence rate each (Table 1). Both studies concluded that the results obtained in Saudi cervical cancer patients are comparable to international rates, namely: (1) the prevalence of HPV infection (89–96%) is in range of the published worldwide estimates of 85–99% (1, 2); and (2) the most common genotypes are the high-risk HPV-16 and -18 that affect together 74.5–78.7% of all HPV-positive patients (Figure 1).

In North America, it was noted that, although there has been significant reduction of the incidence of head and neck cancers as a result of the anti-smoking campaigns since the late 80s, there was a significant increase in the incidence of oropharyngeal squamous cell carcinomas in young (40–55 years) specifically in the tonsils and the base of the tongue where most of these patients are not alcohol or tobacco consumers (33). About 60% of these tumors were found positive for HPV-16, the same type that leads to HPV-associated anogenital cancers. Interestingly, the prevalence of HPV infections in the oral cavity is significantly higher among men than women. In a systematic meta-analysis, Kreimer et al. have reviewed 60 eligible studies that included 5,046 cases of squamous cell carcinomas of the head and neck (34). HPV was prevalent in 35.6% of oropharyngeal, in 23.5% of oral, and in 24% of laryngeal cancers. HPV-16 was by far the commonest subtype in all HPV-positive cancers (87% of oropharyngeal, 68% of oral, and 69% of laryngeal cancers). HPV-18 was the next most common subtype.

**Figure 2:**
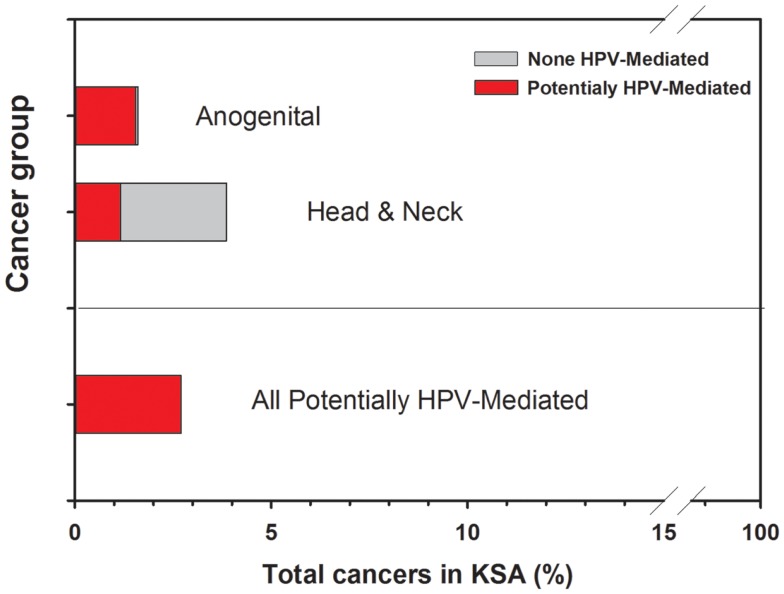
**Burden of potentially HPV-mediated cancers in Saudi Arabia**.

So far, the prevalence of HPV infection in anal, penile, vaginal, vulvar, and oropharyngeal cancers has not yet been explored in Saudi Arabia. Nevertheless, it is important to include these forecasted potential when discussing the eventual impact of HPV vaccines in a society. In fact, the extrapolation of these rough estimated percentages of HPV-positive anogenital (96%) and head and neck (30%) cancers in Saudi Arabia is given in Figure 2. In total, potential HPV-related cancers would represent about 3% of all cancers in both genders. In addition, HPV has more recently been suspected to be implicated in subgroups of colorectal and breast malignancies, however; these studies remain inconclusive, particularly that some experts in the field still do not support such a role in view of the inadequate evidence.

Acknowledging that detection of HPV infection in head and neck cancers remains lacking with no published report to date from Saudi Arabia, few studies had looked at this potential in some other types of tumors such as ocular and prostatic cancers. Karcioglu and Issa had examined the presence of HPV-16/18 in 96 paraffin-embedded external ocular tissues with neoplastic and non-neoplastic lesions and 19 conjunctiva samples free from overt disease (35). They reported HPV positivity in 57% of *in situ* squamous cell carcinoma, in 55% of invasive squamous cell carcinoma, in 20% of climatic droplet keratopathy, in 35% of scarred corneas, and in 32% of normal conjunctival tissue obtained during routine cataract extractions. They concluded that HPV is detectable not only in epithelial neoplasms of the ocular mucous membrane but also in non-neoplastic lesions as well as in apparently healthy conjunctiva. Gazzaz and Mosli had explored the possibility of finding HPV infection in prostatic tissues of 56 patients presenting with benign prostatic hyperplasia (BPH) or prostate cancer (36). The results showed that all the prostatic biopsies were negative for HPV DNA as assessed by the hybrid capture 2 technology that can detect 13 carcinogenic types of HPV infection, and differentiate between the 2 HPV groups, the low and the high/intermediate risk types. The authors concluded that it is unlikely that HPV enhance the risk of prostate cancer.

## Prevention and HPV Vaccines

Prevention of cervical cancer is provided by HPV screening and vaccination, which is an effective measure in many infectious diseases (24, 25). Vaccines were developed against HPV infection to prevent cervical cancer and probably other HPV-related diseases (37). Two types, a bivalent (Cervarix) vaccine that protect against HPV-16 and -18 and a quadrivalent (Gardasil) that is effective against HPV-6, -11, -16, and -18 are being widely introduced in western countries (38, 39), and promising new broad-spectrum HPV vaccines are in development (40). The short term results showed nearly complete efficacy against cervical cytological abnormalities, precancerous lesions, and even genital warts in the case of the quadrivalent vaccine (37, 38).

In principle, the vaccines could be applicable in Saudi Arabia since the incidence of HPV infection in invasive cervical cancer is very high (89–96%) and comparable to the whole-world (85–99%) and that about 75% are HPV-16/18 genotypes (Figure 1), which are covered by both currently available vaccines. Therefore, in theory vaccination is expected to protect against more than three-quarters of cervical cancers in Saudi Arabia as it has been estimated worldwide (38). In contrast, the incidence of cervical cancer is very low in Saudi Arabia, forming only 2.4% of all females’ cancers. From an expenditure point of view, an expert cost-effectiveness analysis that takes into consideration the particular incidence of cervical cancer in the country, is required to justify the implementation of a costly national vaccination program (41). The current information from the SCR and the WHO/ICO indicates that the incidence of cervical cancer in Saudi Arabia is 2.1/100,000 women. This rate is at the threshold of the best performance that cytology screening (Pap smear) can offer and is much lower than the two available vaccines against HPVs can achieve at 9/100,000 for Cervarix and 14/100,000 for Gardasil (37, 38).

Although cost-effectiveness criteria may vary between countries, a preliminary evaluation would suggest that there is no reason for public health to institute either screening or vaccination if Saudi Arabia truly has such a low incidence of cervical cancer. Therefore, theoretically, some might argue that implementing a national vaccination program may not sensibly decrease the incidence of cervical cancer at the population level because it is already very low. In addition, if all 10–14 years old girls are to be vaccinated in Saudi Arabia (1,006,745 girls × 3 shots/girl), this would be a burden on health system and may not be cost-effective in comparison with other health priorities. Nevertheless, there may be specific individual benefits that screening or vaccination may offer for selected women, who would be identified upon careful analysis of the frequency of cervical cancer in groups at risk for HPV infection or women well-informed about the risk and voluntarily wish to be vaccinated. However, taking into consideration the projection of all potentially HPV-associated tumors outlined in Figure 2, in theory, the vaccination is expected to protect about 3% of cancer patients in Saudi Arabia, which would be, from an expenditure standpoint, still require proper cost-effectiveness evaluation in view of the incidence of head and neck [excluding nasopharyngeal carcinoma that would rather be associated with Epstein–Barr virus (EBV) infection] and anogenital cancers in the country. In addition, many DNA vaccines are being developed for the treatment of HPV-16 induced malignancies (42). Most of these vaccines consist of a fusion of E6 or E7 with a “carrier-protein” to generate highly immunogenic E6- or E7-directed DNA vaccines. These vaccines can be used to treat HPV-positive cancers to improve outcome.

Currently, the vaccines are available in major hospitals in Saudi Arabia and are offered with or without fee to requesting girls upon physician’s prescription. Family physicians should also be provided with objective information regarding the HPV vaccine so they would recommend the vaccine to their patients (43). Parents’ involvement is a significant factor in decision making since current practice recommends vaccination to be carried out on minors and young age (9–26 years old) for best efficacy. Hence, educating families and medical staff on the vaccines is important to reconcile with religious values and beliefs bearing in mind that vaccines at large have saved more lives than any medical treatment ever developed.

## Conclusion

Beside the salient difference of having very low incidence of cervical cancer in Saudi Arabia, the involvement of HPV infection in this malignancy is comparable to the rest of the world with HPV-16 and -18 being the two most common genotypes and account together for three-quarters of HPV infection. Although vaccination against HPV would protect three-quarters of cervical cancer patients, the currently reported low incidence and the high cost of the vaccine would make it not-cost-effective in the Kingdom of Saudi Arabia. However, to ascertain or refute these conclusions, further studies are needed to find out the real prevalence of HPV at the population level at large, its association with various types of cancers, and also the impact of local tradition and emerging behavioral trends that could affect HPV transmission.

## Conflict of Interest Statement

The author declares that the research was conducted in the absence of any commercial or financial relationships that could be construed as a potential conflict of interest.
